# An underappreciated DIET for anaerobic petroleum hydrocarbon‐degrading microbial communities

**DOI:** 10.1111/1751-7915.13654

**Published:** 2020-08-30

**Authors:** Federico Aulenta, Matteo Tucci, Carolina Cruz Viggi, Jan Dolfing, Ian M. Head, Amelia‐Elena Rotaru

**Affiliations:** ^1^ Water Research Institute (IRSA) National Research Council (CNR) Monterotondo RM Italy; ^2^ School of Engineering Newcastle University Newcastle upon Tyne UK; ^3^ School of Natural and Environmental Sciences Newcastle University Newcastle upon Tyne UK; ^4^ Department of Biology University of Southern Denmark Odense Denmark

## Abstract

Direct interspecies electron transfer (DIET) via electrically conductive minerals can play a role in the anaerobic oxidation of petroleum hydrocarbons in contaminated sites and can be exploited for the development of new, more effective bioremediation approaches.

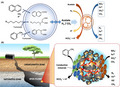

## The discovery of direct interspecies electron transfer

The discovery of direct interspecies electron transfer (DIET) between microbial cells revealed a new and exciting possibility for microbial cooperation in energy‐limited anaerobic ecosystems (Summers *et al*., 2010). The first observation of a DIET association was between two *Geobacter* species, *Geobacter metallireducens* and *Geobacter sulfurreducens,* grown on a medium provided with an electron donor (ethanol) for the first species, and an electron acceptor (fumarate) for the other species. Together the two *Geobacter* formed electrically conductive aggregates. In these aggregates, electrons from the oxidation of the electron donor by *G. metallireducens* were passed to *G. sulfurreducens,* which could use them to reduce fumarate to succinate. The electrical wiring between the two microorganisms was ensured by *c*‐type cytochromes and conductive type IV pili (Summers *et al*., 2010). Pili and *c*‐type cytochromes were only required for DIET associations and unnecessary for associations between *Geobacter* and partners capable of formate or H_2_ transfer (Rotaru *et al*., 2012).

This disruptive discovery shook up a consolidated paradigm according to which the exchange of electrons (as well as of nutrients, carbon substrates and information) among microbes, proceeds via molecular diffusion of soluble (redox‐active) molecules such as H_2_ or formate (Stams and Plugge, 2009; Schink and Stams, 2013). Theoretical analyses indicated that DIET (governed by electrical conductivity) could be substantially faster and more effective than interspecies H_2_ transfer, governed by Fick’s law of molecular diffusion (Cruz Viggi *et al*., 2014; Cheng and Call, 2016). This leads to the intriguing hypothesis that direct electron transfer between microbial species could be much more widespread in natural environments than previously recognized.

Following the proof‐of‐concept study reported by Summers *et al.,* (2010), DIET between a range of microorganisms was explored, most notably between *Geobacter* spp. and methanogens (Rotaru *et al*., 2014; Yee *et al*., 2019; Yee and Rotaru, 2020). Interestingly, several studies showed that DIET interactions could be stimulated by conductive materials (Lovley, 2017). For example, even the addition of relatively small amounts (<50 mg Fe l^−1^) of nano‐sized magnetite nanoparticles could trigger DIET between *Geobacter* species, with a negligible lag phase (You *et al*., 2019). This phenomenon was also observed in syntrophic methanogenic communities (Dubé and Guiot, 2015) and in co‐cultures of *Geobacter sulfurreducens* and *Thiobacillus denitrificans* (Kato *et al*., 2012). It was shown that conductive magnetite particles attached to microbes and served as abiotic electron conduits replacing *c*‐type cytochromes, thus connecting redox reactions catalysed by different species of microorganisms (Liu *et al*., 2015). More recently, a wide range of conductive or semi‐conductive minerals and materials such as pyrite, various types of chars and graphite were found to serve as substitutes for biological connectors to promote and facilitate electron transfer between species (Cheng and Call, 2016).

## Practical implications of DIET in engineered bioprocesses

In the past few years, DIET has been the focus of intense research activities and remarkable efforts have been made to translate such fundamental scientific discoveries into practical strategies to improve engineered bioprocesses. In particular, much attention and research efforts have been dedicated to exploring the use of conductive materials to accelerate and stabilize the anaerobic digestion (AD) of waste organic substrates (Dubé and Guiot, 2015). Indeed, a large number of publications indicated that the addition of electrically conductive particles to anaerobic digestors has the potential to accelerate some of the rate‐limiting steps in the methanogenic conversion of organic substrates, such as the syntrophic oxidation of volatile fatty acids or alcohols (Cruz Viggi *et al*., 2014). The acceleration of syntrophic metabolism by conductive particles is most likely because conductive materials promote higher rates of electron transfer between substrate‐oxidizing *Bacteria* and methanogenic *Archaea* (Morita *et al*., 2011; Liu *et al*., 2012; Martins *et al*., 2018; Park *et al*., 2018). In general, in anaerobic digestors amended with electrically conductive particles, the rate of production and yield of methane were typically higher than in control digestors. Amended digestors were also found to be less susceptible to environmental stresses and/or abrupt changes in operating conditions, such as the applied organic loading rate (Baek *et al*., 2015; Dubé and Guiot, 2015).

The benefits of improving conventional AD processes are clear and are, in part, justified by the rapidly expanding AD market which is being promoted by an increased focus on decarbonizing the energy sector (Edwards *et al*., 2015). Nevertheless, there are many other opportunities and applications for DIET on the horizon. One broad area, which remains largely unexplored, is bioremediation of soil, sediment and groundwater. Here, we point out the role of DIET on the anaerobic biodegradation of petroleum hydrocarbons, driven by naturally occurring or specifically added electro‐conductive minerals or materials. We anticipate that harnessing DIET could contribute to the development of new, more‐effective bioremediation technologies.

## Syntrophic anaerobic biodegradation of hydrocarbon contaminants

Many subsurface environments, such as soils, sediments and aquifers, become rapidly anaerobic after accidental contamination with petroleum hydrocarbons. This is because hydrocarbons serve as electron donors in the metabolism of fast‐growing aerobic microbial communities, thereby leading to a rapid depletion of oxygen, which is only slowly replenished from surrounding oxic environments. Albeit slower than its aerobic counterpart, the biodegradation of a broad variety of aliphatic and aromatic hydrocarbons under anaerobic conditions has been widely documented (Widdel and Rabus, 2001; Gieg *et al*., 2014; Rabus *et al*., 2016). In this context, we expect anaerobic hydrocarbon biodegradation to become an increasingly relevant process in the managed attenuation of contaminated subsurface environments.

**Fig. 1:**
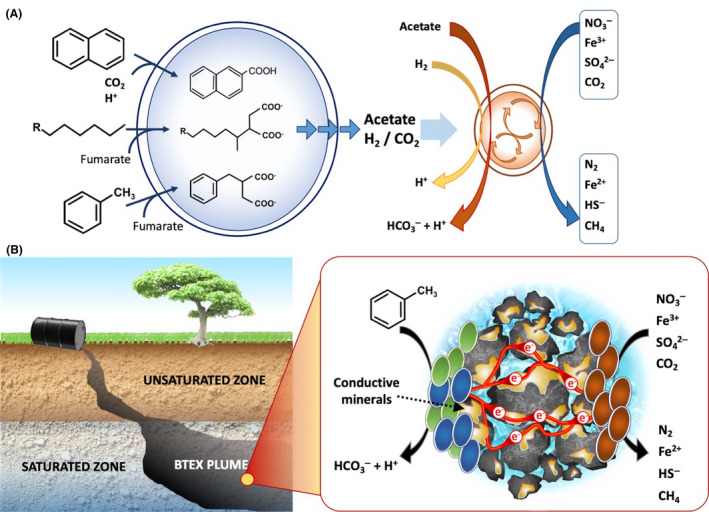
(A) Syntrophic anaerobic degradation of model hydrocarbons under different terminal electron‐accepting conditions. (B) *In situ* syntrophic degradation of toluene driven by direct interspecies electron transfer mediated by conductive minerals.

Under methanogenic conditions, the biodegradation of hydrocarbons requires the syntrophic cooperation of *Bacteria* and *Archaea* (Gieg *et al*., 2014). Following an activation step, typically proceeding via fumarate addition, carboxylation or hydroxylation (Heider, 2007; Meckenstock and Mouttaki, 2011), *Bacteria* catalyse the oxidation of hydrocarbons to CO_2_/H_2_, acetate or a mixture thereof (Fig. 1A). Such reactions are, however, largely endergonic under standard biochemical conditions (e.g. +471 kJ mol^−1^ for hexadecane oxidation to acetate and H_2_), which in turn forces *Bacteria* to rely on the activity of *Archaea* to rapidly convert the produced H_2_ and acetate into methane via exergonic reactions (e.g. −36 kJ mol^−1^ for acetoclastic and −131 kJ mol^−1^ for hydrogenotrophic methanogenesis), thereby making the overall reaction energetically favourable (Dolfing *et al*., 2008). Interestingly, an ever‐increasing number of studies have suggested that syntrophic processes also play a relevant role during biodegradation of hydrocarbons under nitrate‐, iron‐ or sulphate‐reducing conditions (Gieg *et al*., 2014; Fig. 1A). Even the anaerobic oxidation of methane, a primary mechanism for methane removal in ocean sediments, has been found to be driven by the syntrophic partnership between anaerobic methanotrophic (ANME) *Archaea* and deltaproteobacterial sulphate‐reducing bacteria (SRB; Boetius *et al*., 2000; Skennerton *et al*., 2017). The occurrence of all the above described syntrophic microbial relationships has been, however, typically inferred from results of phylogenetic analyses, in some cases corroborated by thermodynamic calculations. Similarly, the electron transfer mechanisms actually involved in the biodegradation processes have been rarely experimentally verified, primarily due to intrinsic methodological difficulties in tracking the involved electron carriers (i.e. H_2_, formate, acetate) transiently accumulating only at extremely low concentration levels and the lack of alternative investigation techniques.

## Hints of DIET‐driven hydrocarbon degradation and urgent research needs

Today, DIET is considered a well‐established metabolic route in the context of anaerobic digestion (Baek *et al*., 2015; Dubé and Guiot, 2015); however, the relevance of this relatively new mechanism in the field of biogeochemistry and/or bioremediation is only marginally explored, in spite of the fact that a few highly relevant publications have clearly pinpointed its likely significance. As an example, recent studies have provided multiple lines of evidence that the syntrophic oxidation of methane (as well as of low molecular weight alkanes, such as butane) by anaerobic methanotrophic *Archaea* (ANME) coupled to the reduction of sulphate by deltaproteobacteria is actually powered by a direct transfer of electrons among the syntrophic partners, occurring via electrical connections based on multihaem cytochromes and/or type IV pili (McGlynn *et al*., 2015; Wegener *et al*., 2015; Chen *et al*., 2019). Activated carbon (AC), an electron conductive material, was found to strongly stimulate (+96%) the biodegradation of naphthalene, a common polycyclic aromatic hydrocarbon (PAH), under anaerobic conditions (Bonaglia *et al*., 2020). By contrast, no stimulatory effects were observed under aerobic conditions, thereby suggesting that AC particles promoted direct interspecies electron transfer (DIET) between microorganisms involved in syntrophic PAH degradation pathways. Rotaru and colleagues (Rotaru *et al*., 2018, 2019) demonstrated that enrichment of microorganisms capable of syntrophic (methanogenic) degradation processes is strictly dependent on the presence of naturally occurring, semi‐conductive iron minerals (e.g. Fe‐oxides and Fe‐sulphides) in sediments from lake La Cruz (Spain) and in coastal sediments of the Baltic Sea. Nielsen and colleagues (Nielsen *et al*., 2010) reported the occurrence of electric currents in marine sediments of Aarhus Bay (DK) which coupled spatially separated redox processes. The authors suggested that semi‐conductive pyrite (i.e. FeS_2_) minerals, highly abundant (up to 30 µmol g^−1^) in such sediments, could be, at least partially, responsible for conducting such electric currents, with these latter being generated by the microbial oxidation of reduced sedimentary organic and inorganic compounds. Moreover, electrically conducting “cable bacteria” similar to those described by Nielsen *et al*. (2010) may have a role in recycling electron acceptors such as sulphate in hydrocarbon contaminated anoxic environments (Müller *et al*., 2016) and thus sustaining anaerobic hydrocarbon degradation.

Electricity spontaneously and widely generated in deep‐sea hydrothermal fields from the microbially driven oxidation of hydrothermal fluids (containing metal ions such as Fe^2+^, Cu^2+^, and reduced gases such as H_2_S, H_2_ and CH_4_) and propagated via sulphide‐rich mineral deposits (Yamamoto *et al*., 2017).

In principle, the enhancement of DIET could also represent a novel bioremediation approach, which might involve the distribution of a colloidal suspension of electrically conductive nanoparticles into the contaminated matrix, so as to create *in situ* a large, electrically conductive and biologically reactive treatment zone (Fig. 1B). An alternative approach may involve devising strategies to stimulate the (biological) formation of conductive particles (e.g. magnetite is an end‐product of the metabolism of iron‐reducing bacteria; iron sulphides can be formed from the reaction of biologically produced sulphide with iron ores) directly in situ. The addition of ethanol, or substrates releasing ethanol upon fermentation, is another interesting strategy which could also be devised to stimulate hydrocarbons biodegradation in subsurface environments, possibly in combination with those previously described. Indeed, recent studies demonstrated that ethanol addition as a co‐substrate during anaerobic degradation of organic substrates triggers DIET in mixed microbial cultures, with this syntrophic/cooperative pathway stably persisting even after ethanol supplementation ceased (Zhao *et al*., 2016, 2017; Zhao and Zhang, 2019). Moreover, the stimulation of methanogenic degradation of organic waste and coal by addition of saturating amounts of the phenazine dye, neutral red, such that filaments of electrically conducting neutral red crystals form, presents an intriguing opportunity for managing direct interspecies electron transfer in natural and engineered environments (Beckmann *et al*., 2016). A similar approach, but involving the use of carbon nanotubes, has also proved effective in triggering DIET‐based syntrophic degradation processes (Salvador *et al*., 2017). Clearly, a critical step towards a deeper comprehension and effective management of DIET‐based biotechnological approaches will necessarily be the development and *ad hoc* application of a broad array of analytical, electrochemical, microscopy and biomolecular techniques specifically designed to identify, monitor and possibly manipulate such tiny electric currents and related chemical reactions, as well as to identify the abundance and expression of genes specifically involved in extracellular electron transfer processes.

In conclusion, it is apparent that a tangible potential exists that hydrocarbon‐degrading microbial communities inhabiting (anthropogenically or naturally) contaminated subsurface environments such as soil, sediments and aquifers can team‐up with a variety of conductive and semi‐conductive particles, abundantly and ubiquitously present in such ecosystems (or ad hoc supplemented), to couple, even spatially distant, biogeochemical reactions and accordingly carry out specific syntrophic and/or cooperative metabolisms of major environmental relevance.
